# Protein interactomes of protein phosphatase 2A B55 regulatory subunits reveal B55-mediated regulation of replication protein A under replication stress

**DOI:** 10.1038/s41598-018-21040-6

**Published:** 2018-02-08

**Authors:** Feifei Wang, Songli Zhu, Laura A. Fisher, Weidong Wang, Gregory G. Oakley, Chunling Li, Aimin Peng

**Affiliations:** 1 Institute of Hypertension, Zhongshan School of Medicine, Sun Yat-sen University, Guangzhou, 510080 China; 2 Department of Oral Biology, College of Dentistry, University of Nebraska Medical Center, Lincoln, NE 68583 USA

## Abstract

The specific function of PP2A, a major serine/threonine phosphatase, is mediated by regulatory targeting subunits, such as members of the B55 family. Although implicated in cell division and other pathways, the specific substrates and functions of B55 targeting subunits are largely undefined. In this study we identified over 100 binding proteins of B55α and B55β in *Xenopus* egg extracts that are involved in metabolism, mitochondria function, molecular trafficking, cell division, cytoskeleton, DNA replication, DNA repair, and cell signaling. Among the B55α and B55β-associated proteins were numerous mitotic regulators, including many substrates of CDK1. Consistently, upregulation of B55α accelerated M-phase exit and inhibited M-phase entry. Moreover, specific substrates of CDK2, including factors of DNA replication and chromatin remodeling were identified within the interactomes of B55α and B55β, suggesting a role for these phosphatase subunits in DNA replication. In particular, we confirmed in human cells that B55α binds RPA and mediates the dephosphorylation of RPA2. The B55-RPA association is disrupted after replication stress, consistent with the induction of RPA2 phosphorylation. Thus, we report here a new mechanism that accounts for both how RPA phosphorylation is modulated by PP2A and how the phosphorylation of RPA2 is abruptly induced after replication stress.

## Introduction

Protein phosphorylation, a major form of post-translational modification, plays a crucial role in regulation of protein functions. The vast majority of protein phosphorylation occurs on specific serine and threonine residues that are oppositely regulated by protein kinases and phosphatases^1^. However, compared to Ser/Thr kinases that are known to play critical roles in numerous cellular processes, Ser/Thr phosphatases are relatively less studied. Ser/Thr phosphatases have been classified into several groups, among which the type 1 (PP1) and type 2A (PP2A) are most abundant^2^. The catalytic subunits of PP1 and PP2A complex with an array of regulatory targeting subunits, which dictate the substrate recognition of the phosphatase holoenzymes. PP2A exists in the cell predominantly as a heterotrimer composed of a catalytic subunit (C), a scaffold subunit (A) and a targeting subunit (B)^3^. The A and C subunits of PP2A each contain two possible variants, whereas the B subunits are encoded by at least 15 genes in mammalian cell. The B subunits are highly diverse in structure, and often classified into 4 groups, including B55/PR55, B56/PR61, PR48/PR72/PR130, and PR93/PR110/striatin^3^.

The B55 group of PP2A regulatory subunits comprises 4 different isoforms (α, β, γ and δ) that share high levels of sequence similarity, but may exhibit distinct patterns of expression and subcellular localization^4^. The yeast homolog of B55, Cdc55, was shown to regulate cell cycle progression, particularly cell division^5,6^. A conserved role of B55 in mitotic regulation was also implicated in vertebrates^7–10^. Moreover, emerging evidence linked B55 to regulation of cell signaling, cytoskeleton, and Golgi dynamics^4,8,10–12^. The B55 subunits are of interests to human cancer, as several genomic studies suggested these subunits as potential tumor suppressors. For example, one study showed depletion of the B55α gene was related to 67% of prostate cancer cases^13^. Moreover, a large-scale genomic and transcriptomic analysis of 2,000 breast tumors identified B55α as one of the most commonly silenced genes; and the subgroup of breast cancer patients with loss of B55α suffered from poor treatment outcome and survival^14^. Other studies associated B55α to childhood teratoma^15^, prostate cancer^16^, colorectal cancer^17^, lung cancer^18^, and leukemia^19^. Gene deletion of B55β was less implicated in cancer, but epigenetic suppression of B55β expression was functionally characterized in breast and colon cancer^20,21^.

As a major group of the PP2A targeting subunits, it is expected that B55 directs PP2A to a large number of substrates. However, to date only a few phosphoproteins were defined as direct substrates of PP2A/B55. To fill in this large gap in knowledge, in the current study we characterize the interactomes of B55α and B55β that each contains over 100 proteins. Among these proteins were factors involved in cell division, DNA repair and replication, components of actin, microtubule, Golgi and nucleopore, and regulators of cellular signaling, metabolism and mitochondria function. A fraction of these proteins were previously known as substrates or interactors of B55, but the majority of them are new discoveries of this study.

Interestingly, our proteomic analysis suggested a role of B55 in regulation of multiple DNA replication proteins, particularly replication protein A (RPA). RPA is a crucial single strand DNA-binding protein complex that orchestrates DNA replication and repair^22–24^. Under conditions of replication stress, the RPA2 subunit of RPA is hyperphosphorylated by CDK2, ATM/ATR, DNA-PK and other kinases. In turn, RPA phosphorylation facilitates the stabilization, repair, and recovery of stalled replication forks^25–27^. Not surprisingly, RPA and its phosphorylation have been implicated as a valuable marker for cancer progression and drug target for cancer therapy^28–30^. Here we show that B55α associates with RPA, and the association is reduced upon replication stress, presumably as a mechanism to allow phosphorylation of RPA2. Consistently, ectopic expression of B55α suppressed RPA phosphorylation, and attenuated checkpoint signaling after replication stress.

## Results

### Characterization of the B55α and B55β interactomes in *Xenopus* egg extracts

We sought to reveal the function of B55 in *Xenopus* egg extract, a well-established cell-free model system in which protein interaction networks and cellular activities are well preserved. For example, the extracts can biochemically undergo multiple rounds of cell cycle progression. In addition, numerous studies illustrated the value of the extracts in recapitulating DNA replication, repair, microtubule assembly, signaling transduction, and apoptosis^31–35^.

**Figure 1:**
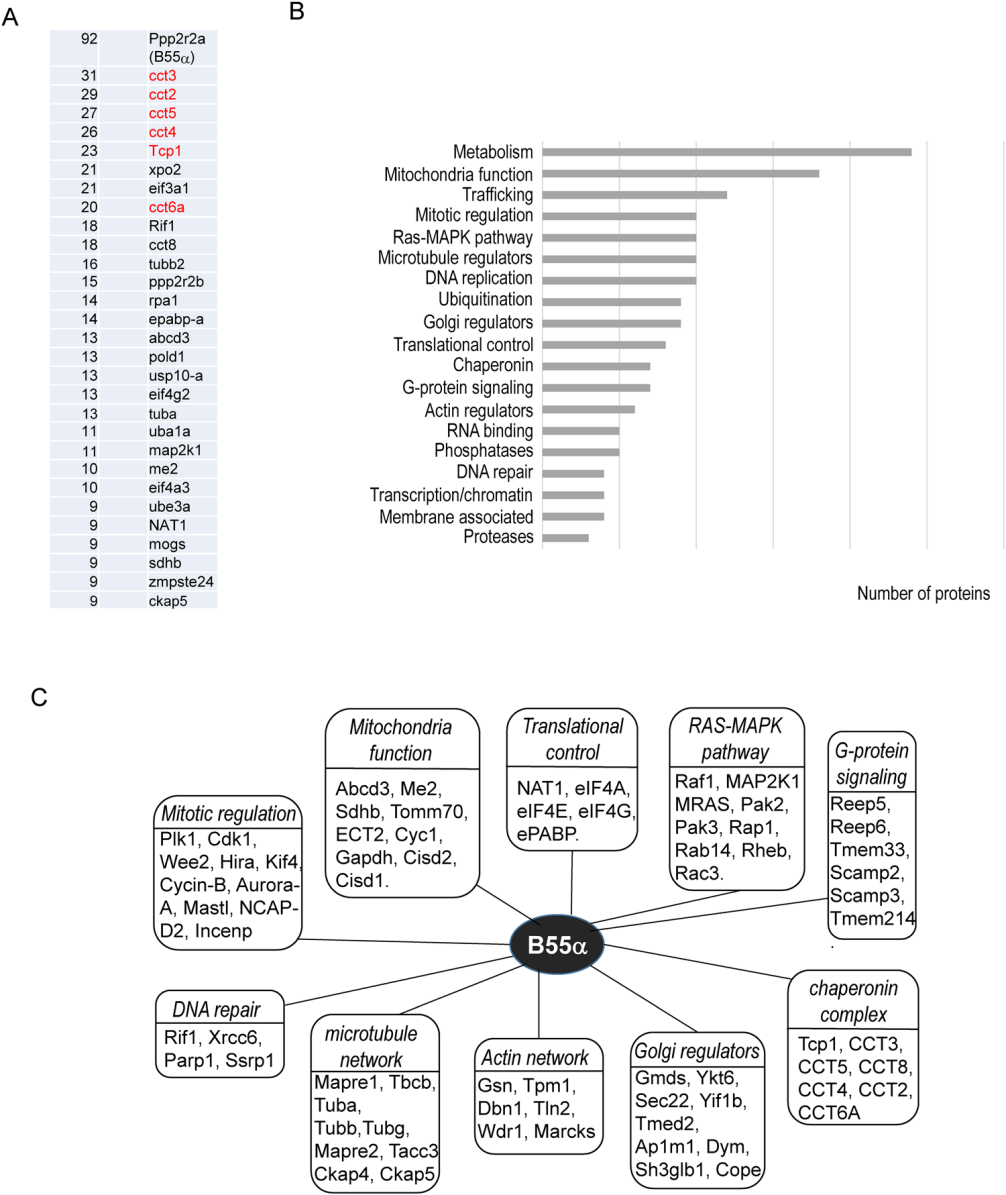
The protein interaction network of B55α. (**A**) The top 30 most abundant proteins identified as B55α-associated proteins. Recombinant B55α was purified, and incubated in interphase *Xenopus* egg extracts for 30 min. B55α-associated protein complexes were then purified and analyzed by mass spectrometry, as described in Materials and Methods. The identified proteins and numbers of peptides are shown. The proteins previously known to bind B55α are shown in red. (**B**) B55α-associated proteins were classified into various functional groups. (**C**) Representative B55α-associated proteins involved in distinct cellular pathways are shown.

We incubated the recombinant B55α or B55β protein in *Xenopus* egg extracts, affinity purified the protein, and analyzed the eluted protein complexes by mass spectrometry. As expected, a large number of peptides from B55α and B55β themselves were recovered, along with numerous co-purified proteins. The raw results were processed to eliminate non-specific binding proteins, as determined by a parallel pull-down experiment using control beads. Moreover, proteins identified by one single peptide were removed to improve stringency. In turn, 176 and 135 proteins were identified as high confidence candidates of the associated proteins of B55α and B55β, respective (Supplementary Tables 1 and 2). The most abundant proteins associated with B55α are components of the chaperonin containing TCP1 complex (CCT), and regulators of DNA replication, DNA repair, mitochondria, and translation (Fig. 1A). Overall, the 176 B55α-associated proteins are involved in a wide range of cellular processes, including cellular metabolic pathways, mitochondria functions, molecular trafficking, cytoskeleton, cell cycle regulation, Golgi regulation, G-protein signaling and nucleic acid metabolism (Fig. 1B,C). This diversity of B55α-associated proteins signals for complex roles of B55α in various cellular processes, which may be significantly underappreciated in the context of existing evidence. Of note, our analysis confirmed the previously implicated connections between B55α and the CCT complex^36^, cell division (such as Plk1, Mastl, and mitotic spindle components)^8,9,37–39^, DNA replication and repair (such as Mcm and Ruvbl)^40,41^, RAS and related small GTPase signaling^36,42^, eukaryotic initiation factor 4F (eIF4F) complex^43^, Golgi and trafficking (such as Ap1m1, and Xpo1)^44,45^.

**Figure 2:**
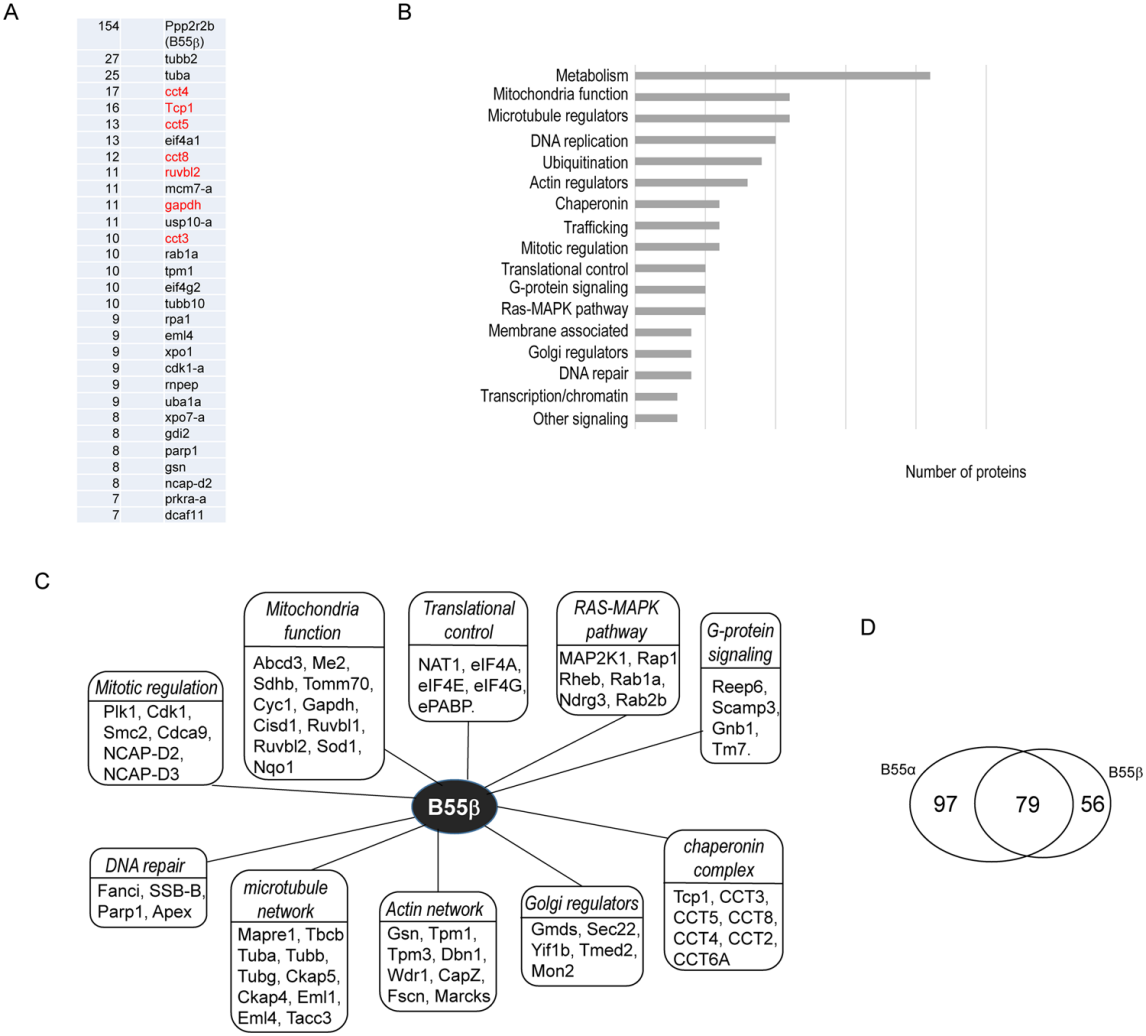
The protein interaction network of B55β. (**A**) The top 30 most abundant proteins identified as B55β-associated proteins. B55β-associated protein complexes were purified and analyzed by mass spectrometry, as described in Materials and Methods. The identified proteins and numbers of peptides are shown. The proteins previously known to bind B55β are shown in red. (**B**) B55β-associated proteins were classified into various functional groups. (**C**) Representative B55β-associated proteins involved in distinct cellular pathways are shown. (**D**) The numbers of common and unique binding proteins of B55α and B55β are shown.

Our results showed that, like B55α, B55β associated with a large number of proteins related to cellular metabolism, mitochondria functions, molecular trafficking, cytoskeleton, cell cycle regulation, Golgi regulation, G-protein signaling and nucleic acid metabolism (Fig. 2A–C, Supplementary Table 2). The top candidates of B55β-associated proteins significantly overlaped with those of B55α, including CCT components, and regulators of DNA replication, DNA repair, mitochondria, and translation (Fig. 2A). In total 79 proteins associated with both B55α and B55β, whereas 97 and 56 proteins appeared as unique binding-proteins of B55α and B55β, respectively. The finding of a large number of isoform-specific binding proteins for B55α and B55β is interesting, given the high level of sequence similarity between these isoforms. It is not well-defined if B55 isoforms recognize different proteins and have distinct functions. This possibility is however often suggested, as a mechanism to achieve the regulatory complexity and substrate specificity of PP2A holoenzymes. Thus, our findings provide potential evidence to support the notion that B55 isoforms, despite sequence similarities, exhibit different affinity toward substrates.

### Validation of B55α and B55β-associated proteins in *Xenopus* egg extracts

**Figure 3:**
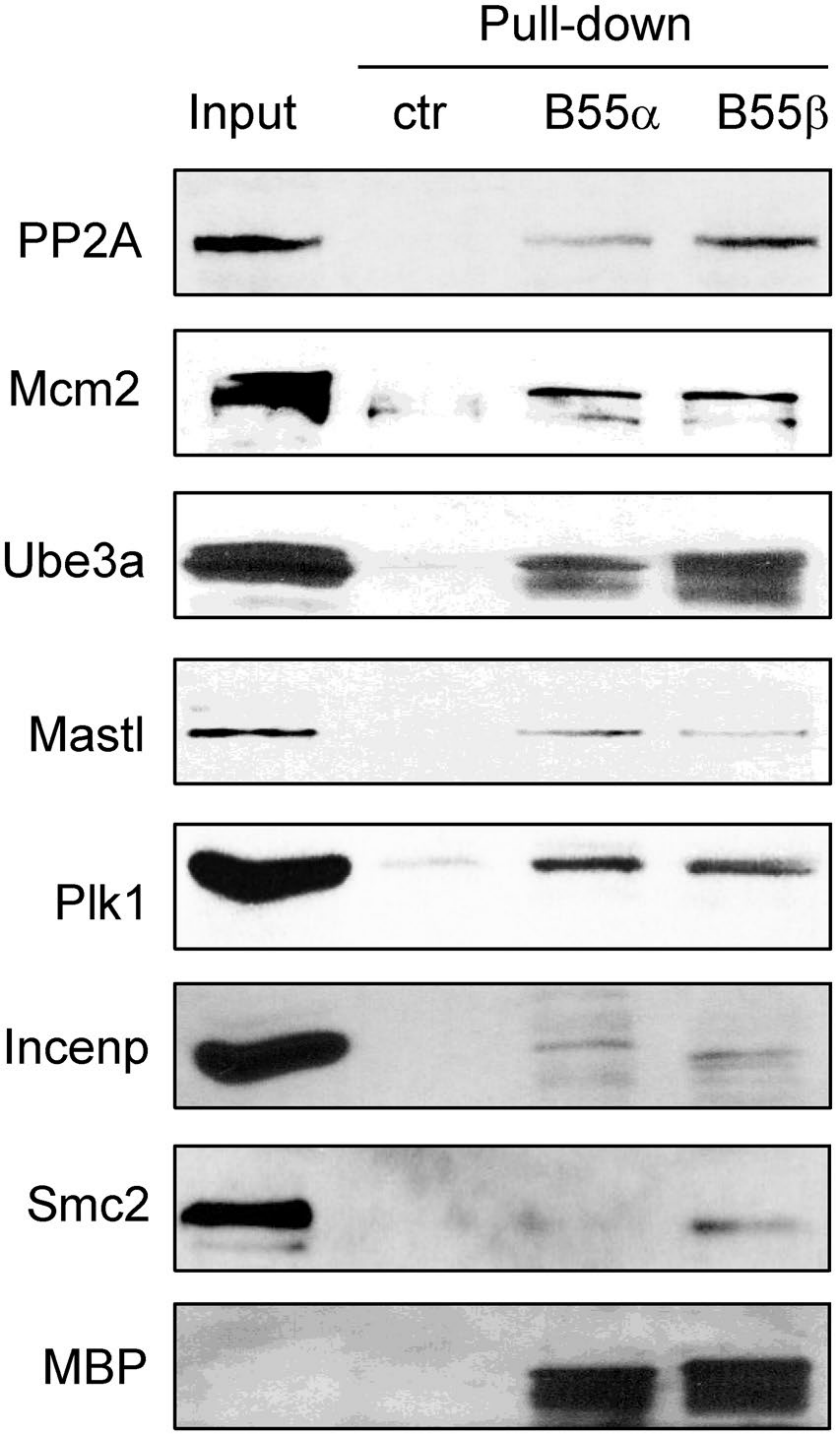
Validation of B55α and B55β-associated proteins. B55α or B55β pull-down was performed in *Xenopus* egg extracts. A control pull-down was performed using the same volume of amylose beads that were not conjugated with proteins. The extract input (approximately 20%), control (ctr) pull-down and B55α or B55β pull-down samples were analyzed by immunoblotting using PP2A, Mcm2, Ube3a, Mastl, Plk1, Incenp, Smc2, and MBP antibodies.

The study identified a number of proteins that are known to bind B55, thus providing a validation for the results. Moreover, as we identified a large number of new proteins associated with B55α and B55β, we sought to confirm some of these candidates by immunoblotting. Importantly, although PP2A was not among the B55-associated proteins identified by mass spectrometry, we confirmed by immunoblotting that both B55α and B55β co-precipitated the catalytic subunit of PP2A (Fig. 3). In addition, DNA replication protein Mcm2, ubiquitin E3 ligase Ube3a, mitotic kinases Mastl and Plk1, and chromosomal passenger complex (CPC) component Incenp were co-purified with B55α and B55β (Fig. 3). Condensin subunit Smc2 was present in the protein complex of B55β but not B55α, consistent with the outcome of mass spectrometry (Supplementary Tables 1 and 2). Overall, we confirmed the association of B55 with several candidates identified in the proteomic analysis. These candidates were selected because they are involved in cell cycle regulation, and we possess specific antibodies that recognize their homologs in *Xenopus*.

### Regulation of the cell cycle and CDK-substrates by B55α and B55β

**Figure 4:**
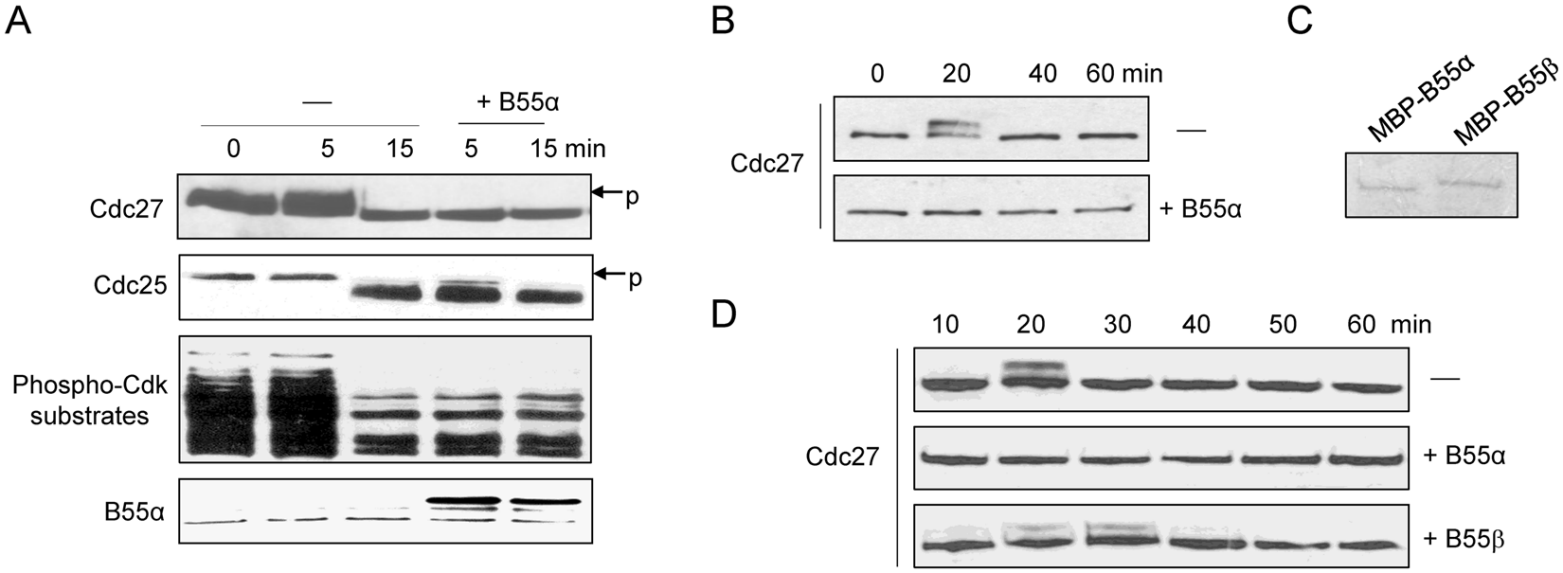
Regulation of the cell cycle by B55α and B55β. (**A**) Metaphase-arrested *Xenopus* egg extracts were supplemented with recombinant B55α or control buffer for 10 min. These extracts were then released into interphase by the addition of Calcium at time 0. The extract samples were collected at the indicated time points and analyzed by immunoblotting. Phosphorylation (band-shift) of Cdc27 and Cdc25, and the global phosphorylation of Cdk substrates are markers of mitosis. (**B**) Interphase *Xenopus* egg extracts were supplemented with recombinant B55α or control buffer. The extract samples were collected at the indicated time points and analyzed by immunoblotting. Mitosis is indicated by Cdc27 phosphorylation. (**C**) MBP-B55α and B55β were purified and examined by Coomassie staining. (**D**) Interphase *Xenopus* egg extracts were supplemented with recombinant B55α, B55β, or control buffer. The extract samples were collected at the indicated time points and analyzed by immunoblotting. Mitosis is indicated by Cdc27 phosphorylation.

Among the associated proteins of B55α and B55β were many cell cycle regulators. In *Xenopus* egg extracts, PP2A/B55δ was characterized as a phosphatase that dephosphorylates mitotic substrates of CDK1^7^. Thus, other B55 subunits, including B55α and B55β, may also act on substrates of CDKs. In fact, a RNAi-based screen in human cells revealed B55α as a regulator of mitotic exit^10^. Here we show that upregulation of B55α in metaphase-arrested *Xenopus* egg extracts accelerated M-phase exit (Fig. 4A), and the addition of B55α in interphase egg extracts suppressed mitotic entry (Fig. 4B). Moreover, although both B55α and B55β inhibited mitotic progression in interphase egg extracts, the anti-mitotic effect appeared less profound for B55β, compared to B55α (Fig. 4C,D).

CDK1 phosphorylates a wide range of substrates to mediate mitotic progression, and the regulated dephosphorylation of CDK substrates allows mitotic exit^46,47^. Various phosphatases, including PP2A, PP1 and Cdc14, were implicated in mitotic exit, but the details about how each phosphatase acts on specific substrates of CDK1 remain to be revealed^46,48–52^. Interestingly, numerous substrates of CDK1 involved in cell division were identified as associated proteins of B55α and B55β (Fig. S1)^53^. For example, B55α and B55β associated with Aurora-B and other members of the CPC complex, multiple subunits of the condensin complex, chromatin remodeling factors, and mitotic spindle components (Fig. S1)^53^. Thus, our findings shed new light on the function and mechanism of B55 in mediating the dephosphorylation of a subset of mitotic phospho-substrates.

In addition to mitotic regulation, our proteomic identification of B55α and B55β-associated proteins revealed a number of proteins involved in DNA replication, including RPA, Cdc6, Cdc45, DNA primase, and the minichromosome maintenance (Mcm) complex. Thus, B55 may play a role in regulation of these DNA replication factors. As many of these replication factors are substrates of CDK2, we speculate that, like in mitotic regulation, B55 may function in DNA replication by dephosphorylating substrates of CDK2. To this end, many known substrates of CDK2, including replication factors, chromatin and ribosome regulators, and cell signaling proteins, are B55α and B55β-associated (Fig. S2).

### Association of B55 with RPA

**Figure 5:**
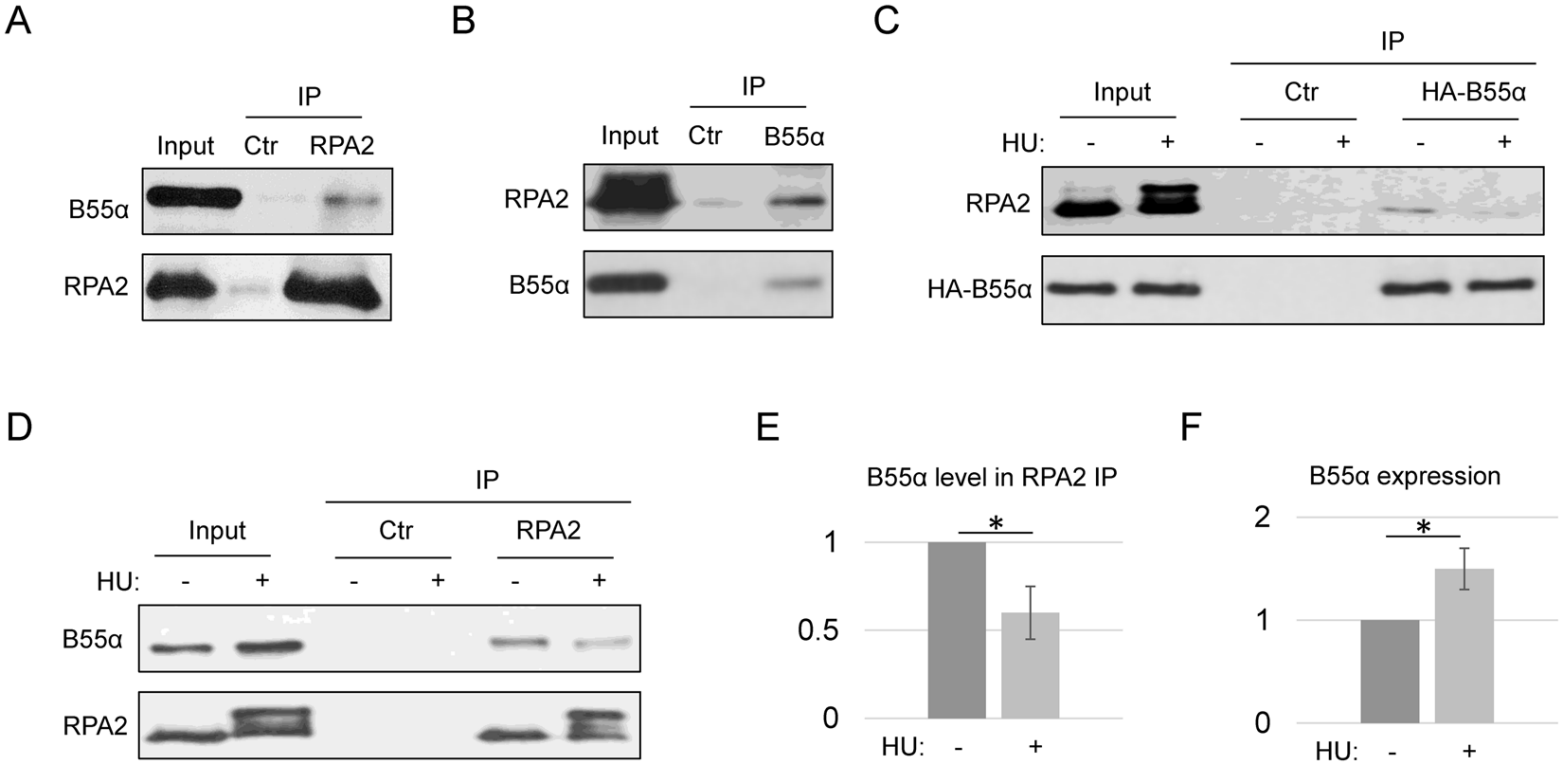
RPA and B55α association. (**A**) RPA2 IP was performed in HeLa cell lysates as described in Material and Methods. The input at 10%, control IP (with blank beads), and RPA2 IP were analyzed by immunoblotting for B55α and RPA2. (**B**) B55α IP was performed in HeLa cell lysates. The input at 10%, control IP (with blank beads), and B55α IP were analyzed by immunoblotting for B55α and RPA2. (**C**) HA-B55α was expressed in HeLa cells, which were treated with or without HU (1 mM, 24 h). HA IP was performed in the cell lysates. The input at 10%, control IP (with blank beads), and B55α IP were analyzed by immunoblotting for B55α and RPA2. (**D**) RPA2 IP was performed in the lysates of HeLa cells that were pre-treated with HU (1 mM, 24 h) or mock-treated. The input at 10%, control IP (with blank beads), and B55α IP were analyzed by immunoblotting for B55α and RPA2. (**E**) The level of B55α in RPA2 IP was examined by immunoblotting and quantified using ImageJ. The mean values and standard deviations were calculated from three independent experiments. Statistical significance was analyzed using an unpaired 2-tailed Student’s t-test. A p-value < 0.05 was considered statistically significant (*). (**F**) The expression level of B55α was examined by immunoblotting and quantified using ImageJ. The mean values and standard deviations were calculated from three independent experiments. Statistical significance was analyzed using an unpaired 2-tailed Student’s t-test. A p-value < 0.05 was considered statistically significant (*).

Our proteomic study revealed RPA as a major associated partner of B55α and B55β. As an essential single strand DNA-binding protein complex, RPA is itself regulated by phosphorylation at several serine/threonine residues within the N-terminus of the RPA2 subunit^22,54^. It has been shown that phosphorylation of RPA is largely dispensable for unperturbed DNA replication, but plays a pivotal role in the cellular response to replication stress and DNA damage^55,56^. Upon replication stress, RPA2 phosphorylation by CDK2 and other kinases facilitates the stabilization, repair, and recovery of stalled replication forks^25–27^. We confirmed the RPA and B55 association in human cells at the endogenous level by reciprocal co-immunoprecipitation (Fig. 5A,B). Notably, although the RPA peptides identified in our initial proteomic study were all derived from RPA1, we believe that B55 associates with the trimeric RPA complex, as evidenced by the co-immunoprecipitation of RPA2 with B55α (Fig. 5A,B).

Next we sought to investigate the impact of replication stress on the RPA and B55 association. The immunoprecipitation of HA-B55α recovered significantly less RPA2 after HU (Fig. 5C). Both hyperphosphorylated and hypophosphorylated forms of RPA2, as judged by gel retardation, exhibited reduced association with B55 (Fig. 5C). Consistently, the immunoprecipitation of RPA2 brought down less amount of B55α after HU (Fig. 5D,E). Interestingly, despite the reduced B55 and RPA association, the total expression level of B55α was moderately elevated upon hydroxyurea (HU)-induced replication stress (Fig. 5D,F), suggesting that B55α plays an active role in the cellular response to replication stress, and that B55 may exhibit an altered spectrum of substrates under replication stress.

### Suppressed RPA phosphorylation by B55α overexpression

**Figure 6:**
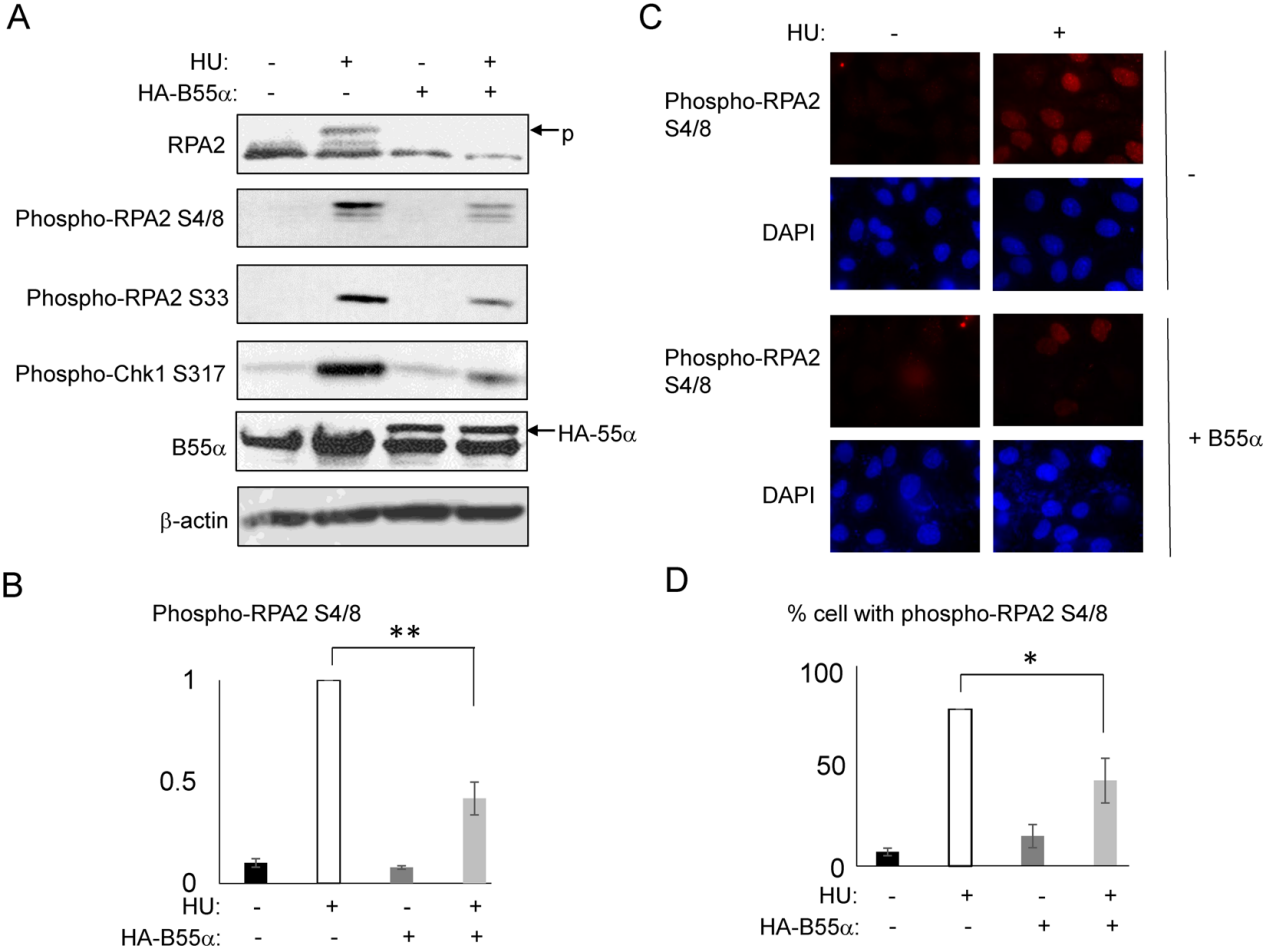
B55α-mediates RPA2 dephosphorylation. (**A**) HeLa cells with or without expression of HA-B55α, were treated with HU (1 mM, 24 h) as indicated. The cell lysates were analyzed by immunoblotting for RPA2, B55α, phospho-RPA2 Ser-4/Ser-8, Ser-33, phospho-Chk1 Ser-317, and β-actin. (**B**) The level of RPA2 S4/8 phosphorylation was examined by immunoblotting, as in panel A, and quantified using ImageJ. The mean values and standard deviations were calculated from three independent experiments. Statistical significance was analyzed using an unpaired 2-tailed Student’s t-test. A p-value < 0.001 was considered statistically highly significant (**). (**C**) HeLa cells were treated with B55α expression and HU, as in panel A. Immunofluorescence was performed using phospho-RPA2 Ser-4/Ser-8 antibody. (**D**) The percentage of cells exhibited positive RPA2 S4/8 phosphorylation was examined by immunofluorescence, as in panel C, and quantified. The mean values and standard deviations were calculated from three independent experiments. Statistical significance was analyzed using an unpaired 2-tailed Student’s t-test. A p-value < 0.05 was considered statistically significant (*).

To confirm the role of B55 in mediating the dephosphorylation of RPA2, we ectopically expressed B55α in human cells, which were then challenged with HU, and analyzed for RPA2 phosphorylation by immunoblotting. As expected, HU-induced RPA phosphorylation at Ser-4/Ser-8 and Ser-33 was substantially reduced in cells harboring B55α overexpression (Fig. 6A,B). The phosphorylation of Chk1, a downstream event of RPA phosphorylation^57^, also partially diminished (Fig. 6A). Similarly, the immunofluorescence analysis confirmed that overexpression of B55α diminished the induction of RPA2 Ser4/Ser-8 phosphorylation after HU (Fig. 6C,D). While these results support a role of B55 in dephosphorylating RPA2, the expression of B55α did not fully suppress RPA2 phosphorylation, potentially due to two reasons: first, our ectopic expression resulted in only approximately 50% increase in the total B55α expression; and second, PP2A/B55 may be only partially responsible for RPA2 dephosphorylation as PP4 was previously known to mediate RPA2 dephosphorylation^58^.

## Discussion

It was established that PP2A dephosphorylates nearly half of all Ser/Thr phospho-residues, and thereby modulating numerous cellular processes. The regulatory complexity and substrate specificity of PP2A at the holoenzyme level is achieved via a variety of distinct PP2A-B, or regulatory substrates, including the B55 group members^4,11^. Although previous studies connected B55 to several substrates, research efforts using systematic approaches may be amenable to substantially advance the understanding of the B55 function. In particular, because the targeting substrates bridge PP2A with specific substrates, the protein interaction network of these targeting subunits can potentially reveal a large number of specific substrates and regulators.

In this study we characterized the protein interactomes of B55α or B55β, two members the PP2A-B family targeting subunits. As a major group of PP2A targeting subunits, B55 is likely to function in a wide range of pathways. For example, the involvement of B55 in cell signaling, cytoskeleton, and Golgi dynamics has been suggested^4,8,10–12^. To better understand the function of B55 in these processes, it is urgent to reveal specific substrates of B55, as well as interacting proteins that modulate the activity of B55. Here we identified over 100 potential binding proteins for B55α and B55β. Proteins associated with B55α or B55β are diversely involved in numerous cellular processes, such as metabolism, cell division, cytoskeleton, DNA replication and repair. Thus, these findings extended the current knowledge about B55-mediated cellular function and processes. Future studies are necessary to clarify if many of these binding proteins are direct substrates of PP2A/B55.

One of the B55-associated proteins identified in this study is RPA. Notably, a previous study showed that PP2A dephosphorylates RPA2^59^, but the underlying targeting subunit remains to be identified. We demonstrated in human cells that B55α binds RPA and mediates the dephosphorylation of RPA2. Interestingly, the B55-RPA association is reduced after replication stress, presumably so as to allow the induction of RPA2 phosphorylation. This finding is of great interest as it argues that the induction of RPA2 phosphorylation after replication stress and DNA damage is at least partially due to the suppression of the phosphatase-mediated RPA2 dephosphorylation. With our discoveries arise several intriguing questions. First, further mechanistic insights are needed to clarify how the RPA2-B55 association is regulated. Second, it remains to be confirmed if PP2A/B55 acts to directly dephosphorylate RPA2. Moreover, as the N-terminus of RPA2 is clustered with many inter-dependent phospho-residues which are targeted by different kinases, it would be necessary to define the specific sites that are dephosphorylated by PP2A/B55. Cundell *et al*.^8^ reported recently that S/TP residues surrounded by two positively-charged basic patches are more likely to under B55-dependent dephosphorylation. However, none of the phospho-residues at the N-terminus of RPA2 matches this structural description. Third, PP4 was also shown to mediate RPA phosphorylation^58^, but it is unclear if this regulation similarly responds to replication stress. Finally, we showed that the expression level of B55α increases after replication stress while its association with RPA is reduced. The increased expression of B55α after HU is a new finding of the study. Presumably, the upregulated B55 plays a role in the cellular responses to replication stress and DNA damage. Along this line, our proteomic analysis revealed multiple DNA repair factors as B55-asscoated proteins, although the precise role of B55 in these processes remain to be investigated. B55 and RPA dissociate after HU, potentially as a mechanism to allow RPA phosphorylation. We reason that HU may alter the substrate recognition of B55, which is released from some substrates but increasingly targeted to others. A similar example is that B55 transiently dissociates from ATM after DNA damage to allow ATM phosphorylation^60^. Therefore, it is interesting to uncover the dynamic function and substrate recognition of PP2A/B55 after replication stress and DNA damage.

## Materials and Methods

### Cell culture and treatment

Human cervix carcinoma (HeLa) cells, authenticated by ATCC, were maintained in Dulbecco’s modified Eagle medium (DMEM, Hyclone) with 10% fetal bovine serum (FBS, Hyclone). The HA-B55α expression vector was a gift from Dr. Xuan Liu (University of California Riverside)^61^, and transfected into cells using Lipofectamine (Thermo Fisher).

### Immunoblotting and immunoprecipitation

Immunoblotting was performed following sodium dodecyl sulfate-polyacrylamide gel electrophoresis (SDS-PAGE), as previously described^62^. Antibodies used in immunoblotting include: Chk1 phospho-S317, Mcm2, Ube3a, Incenp, Smc2 antibodies from Bethyl Laboratories (Montgomery, TX); B55α antibody from Abcam (Cambridge, MA); β-actin, phospho-Cdk substrate and Plk1 antibodies from Cell Signaling Technology (Beverly, MA); Mastl antibody from Millipore (Billerica, MA), and phospho-RPA S4/8 and S33 antibodies as previously characterized^57^. *Xenopus* Cdc25 antibody was a gift from Drs. Kumagai and Dunphy (Caltech). For immunoprecipitation, anti-mouse or anti-rabbit magnetic beads (New England Biolabs) were conjugated to primary antibodies, and then incubated in cell lysates for 1 h. The beads were collected using a magnet, washed, eluted with Laemmli sample buffer, and analyzed by immunoblotting.

### Immunofluorescence

Immunofluorescence was performed as previous described^63^. Briefly, cells were fixed in 3% formaldehyde with 0.1% Triton X-100, washed, and blocked in 10% goat serum in PBS. The primary antibody to RPA2 phospho-S4/8 was diluted in the blocking buffer, and incubated with the cells for 2 h. The cells were then incubated with the Alexa Fluor 555 secondary antibody (Invitrogen, 1: 2,000) for 1 h. Imaging was performed using a Zeiss Axiovert 200M inverted fluorescence microscope at the UNMC Advanced Microscopy Core Facility.

### Protein expression, pull-down and mass spectrometry analysis

B55α and B55β were cloned from a *Xenopus* oocyte cDNA library, and then inserted into pMBP-parallel vector with an N-terminal MBP-tag. The recombinant proteins were expressed in BL21 bacterial cells and purified on amylose beads. For the pull-down assay, approximately 10 μg MBP-B55 proteins conjugated on amylose beads (20 μl) were incubated in interphase *Xenopus* egg extracts (40 μl). After 30 min incubation at room temperature, the beads were re-isolated, washed, eluted and then resolved by SDS-PAGE for immunoblotting or mass spectrometry (Taplin mass spectrometry facility, Harvard). The control pull-down was performed using the same volume of amylose beads that were not conjugated with proteins.

### *Xenopus* egg extracts

*Xenopus* egg extracts were prepared as previously described^64^. For the metaphase-arrested cytostatic factor (CSF) extracts, Eggs were treated with 2% cysteine, washed, and then crushed by centrifugation at 10,000 g. The cytoplasmic layer was collected for further experiments. For the interphase, cycling extracts, eggs were treated with 2% cysteine, and then incubated with Ca^2+^ ionophore. The eggs were then washed, and crushed by centrifugation at 10,000 g. The cytoplasmic layer was collected and supplemented with energy mix (7.5 mM creatine phosphate, 1 mM ATP, 1 MgCl_2_).

### Data availability

All data generated or analyzed during this study are included in this published article (and its Supplementary Information Files).

## Electronic supplementary material

Supplementary Information
