# Supplementary material for: Protein interactomes of protein phosphatase 2A B55 regulatory subunits reveal B55-mediated regulation of replication protein A under replication stress
Source: Sci Rep. 2018 Feb 8;8:2683. doi: 10.1038/s41598-018-21040-6 (PMC5805732; doi:10.1038/s41598-018-21040-6)
Supplement: Supplementary file 1 — Supplementary Information [file 41598_2018_21040_MOESM1_ESM.pdf]

Protein interactomes of protein phosphatase 2A B55 regulatory subunits reveal B55-mediated regulation of replication protein A under replication stress

Feifei Wang[1,2], Songli Zhu[2], Laura A. Fisher[2], Weidong Wang[1], Gregory G. Oakley[2], Chunling Li[1]*, and Aimin Peng[2]*

[1.] Institute of Hypertension, Zhongshan School of Medicine, Sun Yat-sen University, Guangzhou, China. 510080.

[2.] Department of Oral Biology, College of Dentistry, University of Nebraska Medical Center, Lincoln, NE 68583.

* To whom reprint requests should be sent: Aimin Peng, Ph.D. Department of Oral Biology, College of Dentistry, University of Nebraska Medical Center, Lincoln, NE 68583. Tel: 402-472-5903, Fax: 402-472-2551. Email: Aimin.Peng@UNMC.edu; Chunling Li, Institute of Hypertension, Zhongshan School of Medicine, Sun Yat-sen University, 74 Zhongshan 2nd Rd., Guangzhou 510080, China. Email: lichl3@mail.sysu.edu.cn.

Supplemental Materials:

Table 1. The proteins associated with B55α.

Table 2. The proteins associated with B55β.

Figure S1. B55α and B55β associate with many mitotic regulators that are substrates of CDK1.

Figure S2. B55α and B55β associate with many substrates of CDK2.

Figure S3. Images of immunoblots.

Table 1
The proteins associated with B55α

#peptide	protein
92	ppp2r2a
31	cct3
29	cct2
27	cct5
26	cct4
23	Tcp1
21	xpo2
21	eif3a1
20	cct6a
18	Rif1
18	cct8
16	tubb2
15	ppp2r2b
14	rpa1
14	epabp-a
13	abcd3
13	pold1
13	usp10-a
13	eif4g2
13	tuba
11	uba1a
11	map2k1
10	me2
10	eif4a3
9	ube3a
9	NAT1
9	mogs
9	sdhb
9	zmpste24
9	ckap5
8	acox2
8	mapre1
8	eif4e1
7	parp1
7	pcbp3
7	lnpep
7	plk1
7	prkra-a
7	cdk1-a
7	tomm70a
7	gsn
7	xrcc6

7	epcam
7	tubb4
6	Ruvbl2
6	mcm2
6	dhrs12
6	dao
6	cyc1
6	eif4e3
5	epm2a
5	hibch
5	rab14
5	gapdh
5	nudt19
5	tpm1
5	mcm7-a
5	ykt6-a
5	ctsd
5	asna1
5	cisd2-a
5	PPP2r2d
5	cul3
5	seh1l-a
5	epabp-b
5	ruvbl1
5	gmds
5	Tacc3
4	sec22b
4	sall4
4	cdc7
4	pold2
4	mcm4-a
4	lypla2
4	vps4b
4	hcfc1
4	rheb
4	ssrp1
4	reep5
4	scamp3
4	ckap4
3	Xpo1
3	pex16
3	wee2-a
3	scamp2
3	kdsr
3	serhl2
3	hira
3	dbn1

3	yif1b-a
3	cops4
3	kif4
3	mboat7
3	vta1
3	sh3glb1
3	pipox
3	tmed2
3	dpm1
3	mcm5-a
3	mcm7-b
3	pbdc1
3	cbs
3	pex1
3	rac3
3	abce1
3	vamp2
3	sod1-a
3	surf4.1
3	spsb1
3	ccnb1
3	etfb
3	Xtr
3	tp53i11
3	reep6
3	tmem33
3	rap1b
2	raf1
2	upk3a
2	tln2
2	ywhaz
2	sigmar1
2	ppp1r3b-b
2	ECT2
2	ctps1-a
2	mras
2	ahsa1
2	tbcb
2	picalm.2
2	Tm7
2	cisd1
2	cgn
2	ap1m1
2	pxmp2
2	nsdhl
2	vamp7
2	prkag2

2	wdr1-a
2	sgpl1
2	xpo7-a
2	ctbs
2	aurkb-a
2	MARCKS
2	ociad1
2	got1
2	sec31b
2	sympk
2	bpnt1
2	pcna
2	dym
2	mastl
2	mri1
2	tsn
2	p4ha2
2	sirt2
2	cops6
2	tubg1
2	tia1
2	uroc1
2	nipsnap1
2	pitpnb
2	tomm40
2	mapre2
2	cope
2	itgb1-a
2	cul4b
2	ik
2	ufd1l
2	cldna
2	dhrs7
2	pak2
2	pak3
2	sri
2	ncap-d2
2	Incenp
2	Nogo
2	tmem214

Table 2
The proteins associated with B55β.

#peptide	protein
154	ppp2r2b
27	tubb2
25	tuba
17	cct4
16	Tcp1
13	cct5
13	eif4a1
12	cct8
11	ruvbl2
11	mcm7-a
11	gapdh
11	usp10-a
10	cct3
10	rab1a
10	tpm1
10	eif4g2
10	tubb10
9	rpa1
9	eml4
9	xpo1
9	cdk1-a
9	rnpep
9	uba1a
8	xpo7-a
8	gdi2
8	parp1
8	gsn
8	ncap-d2
7	prkra-a
7	dcaf11
7	ctbs
6	TPM3
6	wdr1-a
6	prim1
6	ctnnd1
6	cct6a
6	pcna
6	cab39l
6	got1
6	Tm7
6	ruvbl1
6	tuba3

5	mon2
5	ppp2r2a
5	eif4e1
4	ndrg3
4	pold1
4	vta1
4	mcm7-b
4	capzb
4	sh3glb1
4	mcm4-a
4	fanci
4	rab2b
4	tmem14c
4	mri1
4	sod1-a
4	ctsd
4	cyc1
4	dhrs12
4	etfb
4	nipsnap1
4	gmds
4	tuba1
3	anxa4
3	smc2
3	rtca
3	fscn
3	cpvl
3	mcm2
3	taldo1
3	naa40
3	rheb
3	tkt
3	ssb-b
3	cdc45
3	ISWI
3	dbn1
3	vamp2
3	sod1-b
3	eif4g1
3	me2
3	nat1
3	sec22b
3	sall4
3	rheb
3	dpm1
3	map2k1
3	tubg1

3	reep6
3	sdhb
3	dhrs13
2	cdca9
2	nqo1
2	klhdc10
2	cisd1
2	tbcb
2	14-3-3
2	mapre1
2	upk3a
2	pgam1
2	epcam
2	mcm5-a
2	plk1
2	atic
2	pgm1
2	ugdh
2	idh1
2	eml1
2	sae1
2	fbxw1
2	csnk2a2
2	fam115
2	ywhaz
2	ywhaq
2	cul4b
2	ik
2	sri
2	rapl1
2	mogs
2	tomm70a
2	hibch
2	nudt19
2	kdsr
2	tmed2
2	apex1
2	rap1b
2	upk3a
2	sirt2
2	ncap-d3
2	ckap5
2	ppp2cb
2	tmem126a
2	marcks
2	tacc3

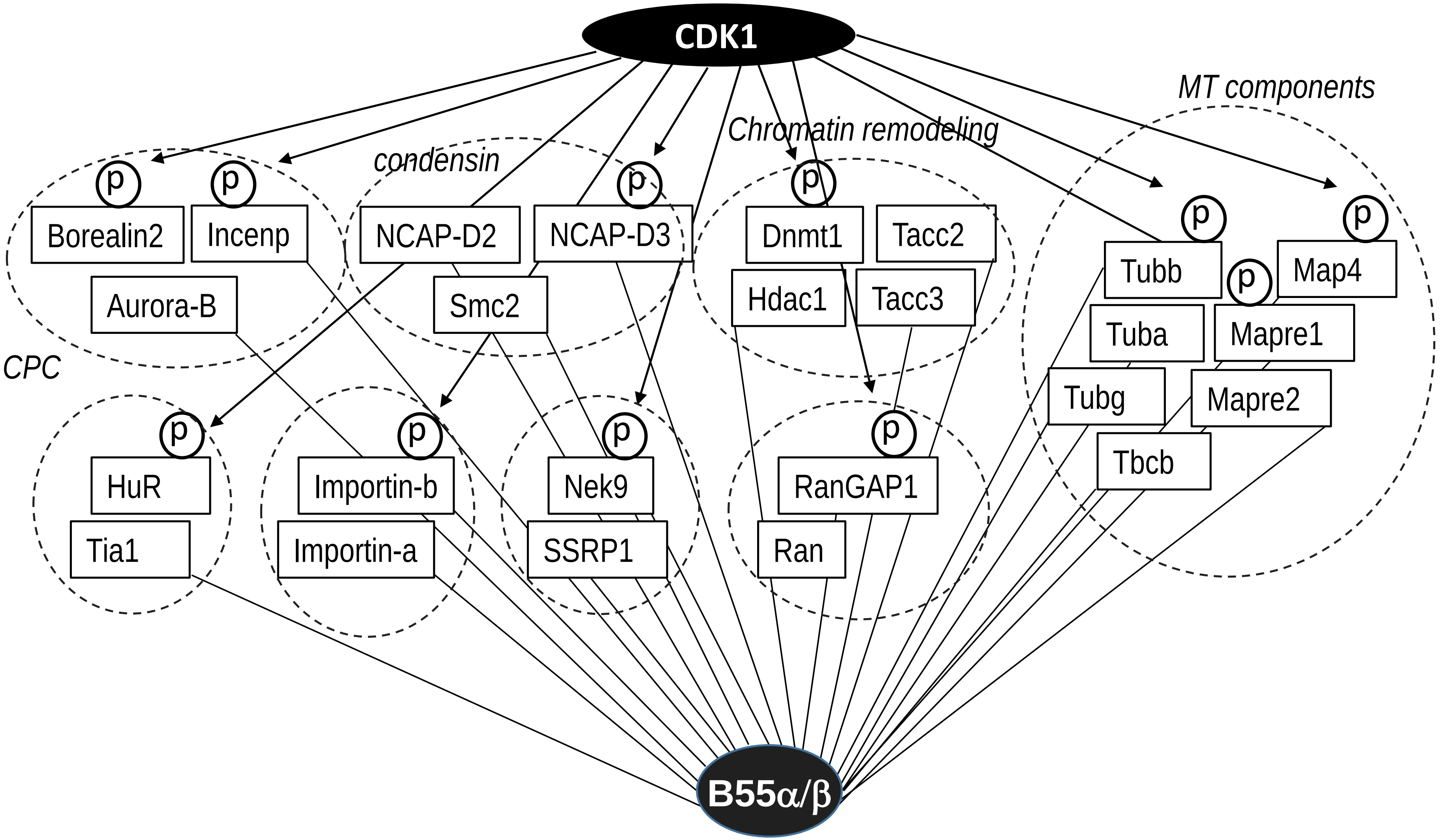

Fig S1. B55α and B55β associate with many mitotic regulators that are substrates of CDK1. B55α and B55β associate with many mitotic regulators that are substrates of CDK1, including components of CPC, condensing, chromatin remodeling complex, microtubule (MT), Importin, Ran GTP system, RNA processing machinery (HuR and Tia1), and FACT (for facilitates chromatin transcription) complex (Nek9 and SSRP1).

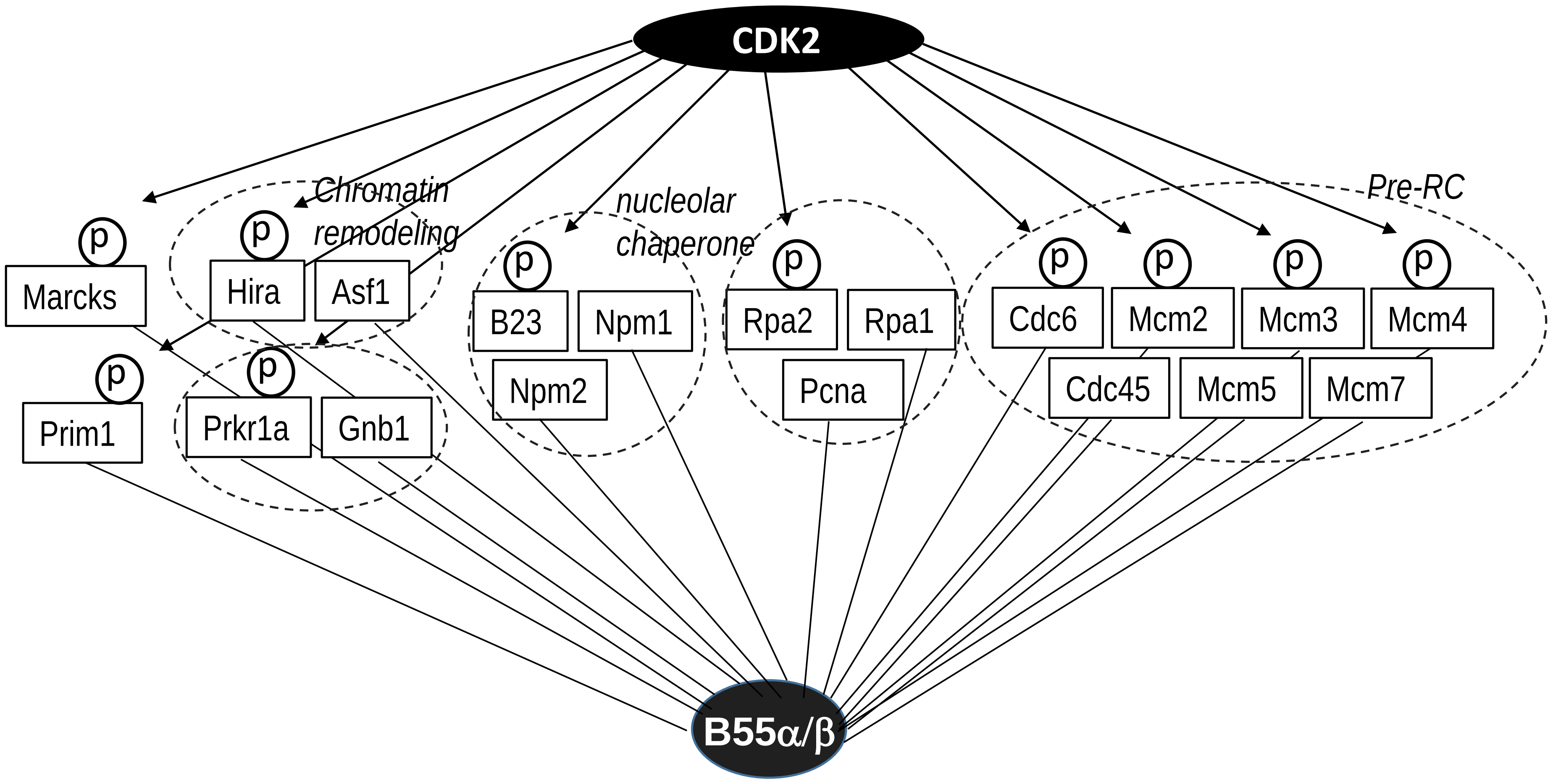

Fig S2. B55α and B55β associate with many substrates of CDK2. B55α and B55β associate with many substrates of CDK2, including components of pre-replication complex (pre-RC), replication machinery (Rpa, Pcna and Prim1), chromatin remodeling complex, nucleolar chaperone, and cell signaling.

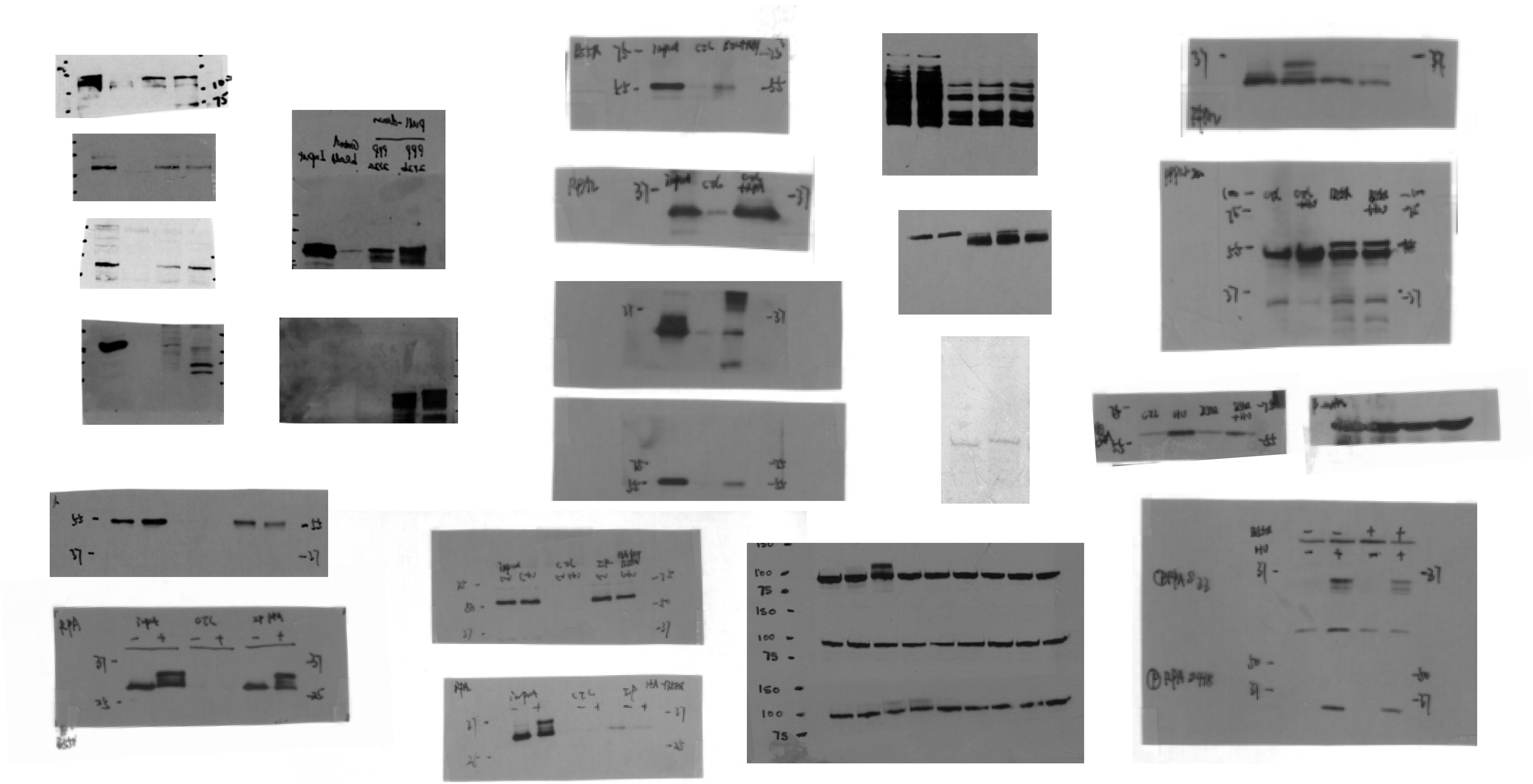

Fig S3. Images of immunoblots.
